# Bibliometric Profile of Global Microplastics Research from 2004 to 2019

**DOI:** 10.3390/ijerph17165639

**Published:** 2020-08-05

**Authors:** Fen Qin, Jing Du, Jian Gao, Guiying Liu, Yonggang Song, Aifu Yang, Hong Wang, Yuan Ding, Qian Wang

**Affiliations:** 1 Dalian University of Technology Library, 2# Linggong Road, Ganjingzi District, Dalian 116024, China; qinfen@dlut.edu.cn (F.Q.); gaojian@dlut.edu.cn (J.G.); wanghong@dlut.edu.cn (H.W.); dingyuan@dlut.edu.cn (Y.D.); 2 Liaoning Ocean and Fisheries Science Research Institute, 50# Heishijiao Road, Shahekou District, Dalian 116023, China; liuguiying2006@126.com (G.L.); hyzjs_lnshky@163.com (Y.S.); 3 Technology Center of Dalian Customs District, 60# Changjiang East Road, Zhongshan District, Dalian 116001, China; yaf_dlhg@hotmail.com; 4 Ocean University of China, 238# Songling Road, Laoshan District, Qiangdao 266100, China; wq13864223681@163.com

**Keywords:** microplastics, bibliometric, network analysis, VOSviewer software, research hotspots

## Abstract

Microplastics (MPs) have generated worldwide attention due to their global distribution in the environment, and their potential harmful effects on human and animal health. To analyze MPs-related scientific publications from a global point of view, we created a bibliometric profile, by searching the Web of Science Core Collection database for the topic “microplastic* or (micro near/1 plastic*)”, in publications dated from 2004 to 2019. The results revealed an increasing trend in publication output, and identified contributions of different countries and their collaborations, as well as influential authors and productive journals in the field of MPs research. Using co-citation network analysis in VOSviewer, we mined cited references for knowledge bases about analytical methods, potential sources and spatial distributions of MPs, the impacts of MPs on organisms, and the interaction of MPs with contaminants, as well as microorganisms. We also identified four global hotspots for MPs related research, using author keywords co-occurrence network analysis of all extracted publications, as well as Essential Science Indicators highly cited papers from Clarivate Analytics. Results of this study provide a valuable reference for ongoing MPs-related research, which may be of intrigue and awesome noteworthiness for relevant researchers.

## 1. Introduction

Plastics used in our daily life and in a wide range of manufacturing processes provide numerous societal benefits, due to their lightweight, durable, and economic nature [1]. However, plastics are resistant to aging, and their refractory degradation makes plastic waste a serious environmental issue [2,3,4]. Microplastics (MPs), which are smaller items of plastic litter, are of increasing concern due to their ubiquitous global distribution in aquatic environments [5,6,7], and their close interactions with biota [8]. Although no universal definition of MP size exists, a diameter smaller than 5 mm is commonly accepted [9]. Examples of MPs include resin pellets, microbeads used for cosmetics or associated with industrial spillages (primary source) [10,11,12], or pieces broken off of larger plastic litter by ultraviolet radiation, oxidation, or mechanical abrasion (secondary source) [13]. Release of synthetic fibers by textile washing is another potential source of MPs [14].

As there has been increasing concern about MPs research, scholars have reviewed literatures in this domain covering different aspects. Initially, reviews of MPs research focused mainly on the marine environment. For example, Cole et al. discussed the sources and transfer of MPs into the marine environment, and assessed the spatial and temporal distribution of MPs in the worldwide marine environment [3], and they concluded that the fate of these MPs was still elusive. Wright et al. investigated the impacts of MPs on marine invertebrates [8]. Later, Horton et al. critically reviewed the presence, behavior, and fate of MPs in terrestrial environments, by evaluating studies of the extent of MPs pollution in freshwater, treated water sources, and even agriculture soil [15]. Recently, the biological effects of MPs have emerged as areas of interest. Researchers have summarized the potential health effects of MPs present in the food chain [16], and emphasized the interaction between MPs and microorganisms [17]. The authors of these reviews amassed, summarized, and extended the MPs-related research based on their long-term research experiences. To date, studies of the evolution of MPs-related scientific research from a global point of view over time were still insufficient. Bibliometric analysis, which takes advantage of bibliometric theory using mathematical and statistical approaches, is a method that can be used to address this knowledge gap. It has been applied to analyze pertinent literatures in various research fields [18,19,20], including environment-related fields [21]. With regard to MPs research, Ivar do Sul et al. summarized the common denominator between MPs and microbiology, using the bibliometric approach [22], and Barboza et al. evaluated research trends and future perspectives on MPs in the marine environment for the period 2004–2014, using the cross-disciplinary quantitative analysis method [23]. As MPs research has increased substantially since 2011, Zhang et al. conducted an in-depth statistical analysis of global MPs research, using the number of publications as a primary metric for productivity of countries, institution, authors and journals [24]. An up-to-date comprehensive review of the scientific literature, which interprets the influence and importance of different countries, authors and journals, as well as co-occurrence keywords analysis initiated in both extracted literatures from the database and Essential Science Indicators (ESI) highly cited papers from Clarivate Analytics; this is still needed to trace global research hotspots in MPs research.

In this study, we conducted an integrated bibliometric analysis of the literatures on MPs research published from 2004 to 2019. The initial time was set as 2004, because that year, Thompson et al. [25] coined the term “microplastics (MPs)” to define the smaller plastic litter. We used the analysis to identify influential countries, international collaborations, contributing authors, preferred journals, a knowledge base of MPs studies, and research hotspots. The results of our analysis provide a valuable picture of the status of current global MP research, and help illuminate the next steps for future studies.

## 2. Materials and Methods

### 2.1. Data Sources

The Web of Science Core Collection (WoSCC), which generates standardized and high-quality academic publication information, is used extensively for the bibliometric examination of the evolution of scientific issues [18,26,27]. On March 12, 2020, all original data were extracted from the online version of the WoSCC database (indexes: Science Citation Index-Expanded and Conference Proceeding Citation Index), using the TOPIC “microplastic* or (micro near/1 plastic*)” for the years 2004 to 2019. ESI highly cited individuals along with the number of their MPs-related publications and ESI highly cited papers were collected by the same TOPIC from Clarivate Analytics on the same day.

### 2.2. Data Screening

Initially, 3246 publications (after removal of duplications) were extracted using our data searching strategy, including some articles related to material science studies. The latter publications could not be removed simply by excluding some keywords in the data search (e.g., by using “not ‘micro-plastic deformation behavior’” or “not ‘micro-plasticity’”), because some studies of biodegradable polymers relate to both material science (composites modification) and our study objective (safe for environment), such as [28]. Thus, we conducted content analyses of titles and abstracts of all 3246 publications, and sometimes the full manuscripts were evaluated to exclude irrelevant publications. Ultimately, 2637 publications written in English or with an English abstract remained after the manual screening of four types of documents (articles, reviews, proceeding papers and book chapters). Because these types of documents contained novel concepts, none of them were excluded from our analysis, thus, these 2637 publications were all included in the bibliometric analysis. Moreover, a total of 395 ESI highly cited MPs-related papers were extracted from Clarivate Analytics.

### 2.3. Analytical Methods

#### 2.3.1. Basic Bibliometric Analysis Method

The basic bibliometric analysis method used a range of indicators to identify distributed characteristics and structural patterns of the general bibliographic data for MPs ongoing work. For example, the year-wise distribution of research output demonstrated the developing trend of increasing work in the MPs discipline. The contribution of an individual country/academic researcher in the MPs scientific research field was ranked by how many times their publication was cited by others (non-self-citation, NSC), and other data recorded included their total number of publications (TNP), sum of times cited (STC), non-self-citation ratio (NSCR), number of publications cited by more than 100 and 50 times, and number of ESI highly cited publications. Preferred journals were identified as those that delivered academic articles and contributed to the development of the research field [29]. Both the journal impact factor (IF) and quartiles in relevant categories were derived from Journal Citation Report (JCR) 2018, and used to explore the publishing journal’s influence in the MPs field and their interdisciplinary research areas. All bibliographic data were analyzed using Microsoft Excel 2016, and figures were created using GraphPad Prism (version 7.04, GraphPad Software Inc., San Diego, CA, USA) and VOSviewer software (version 1.6.9, Centre for Science and Technology Studies, Leiden University, Leiden, The Netherlands). We used the results of quantitative analysis of the evolution of literature, as well as the bibliometric indicators, to present a general informative overview of MPs research during the study time period.

#### 2.3.2. Network Analysis Methods

VOSviewer is a free software tool based on the Java environment, that is suitable for constructing complex networks using large-scale data. Therefore, we used VOSviewer software (version 1.6.9) to conduct an in-depth network analysis to visualize the connections between various MPs-related items, and to explain their network structure.

Countries co-authorship network: We conducted the co-authorship analysis to identify collaboration networks among different countries in the MPs research field. The nodes represented countries contributing to MPs research, and the links between items implied cooperative relationships. The size of the node increased as the number of articles published by an individual country increased. The value of the links indicated the number of times a given country shared co-authorship with others. The strength of the link increased as the number of co-authorships increased.

Cited reference co-citation network: Analyzing a knowledge base in a certain research field can be conducted by co-citation network analysis for the cited references [18]. In our co-citation analysis, the nodes represented scientific references. The node size represented the number of times a reference was cited. The distance between two references indicated the correlation of the articles according to co-citation links, based on the assumption that more frequently co-cited references exhibited greater co-citation strength.

Author keywords co-occurrence network: The keywords that authors provided for their articles about MPs research represented their academic viewpoints. Thus, our author keywords co-occurrence analysis identified important terms in the MPs academic, as well as the research hotspots in the MPs discipline. The nodes represented high-frequency author keywords, and the size of an individual node represented how many times that keyword occurred. The link strength between two nodes indicated the number of articles in which two keywords occurred together.

## 3. Results and Discussion

### 3.1. Basic Bibliometric Analysis

#### 3.1.1. Characteristics of Publication Output

**Figure 1:**
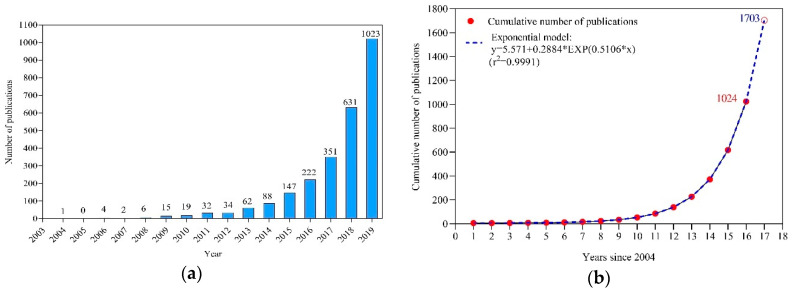
(**a**) Annual number of publications on microplastics (MPs) research from 2004 to 2019, retrieved from WOSCC; (**b**) The cumulative annual number of publications since 2004 follows an exponential model.

The year-wise distribution of publication output revealed the progress of MPs research over time (Figure 1a). The number of publications related to MPs fluctuated slightly from 2004 to 2008, and the annual publications were all less than 100 until 2014. Obvious growth of MPs research began in 2014, when the first United Nations Environment Assembly of the United Nations Environment Programme (UNEP) issued the resolution UNEP/EA.1/L.8, which emphasized critical activities to address marine plastic debris and MPs challenges. Governments worldwide began a shared commitment to addressing MPs problems and to conducting the systematic research of many aspects of MPs [30,31,32,33]. The cumulative number of annual publications since 2004 follows an exponential model (Figure 1b), and the simulation results suggest that publications about MPs issues might increase to 1703 in 2020. On May 8, 2020, we collected data using the same TOPIC from the WoSCC database, for the time period spanning January 1, 2020 to April 30, 2020, and identified 442 relevant publications. This value was less than the expected value for one-third of the year 2020 (567), which may be a result of the significant disruption that is being caused by the COVID-19 pandemic.

#### 3.1.2. National Contribution Analysis

According to the author address information in the 2637 publications, 104 countries made contributions to MPs research during the study time period. Table 1 lists the top 10 countries by their NSC times of publications in MPs research field, as well as TNP (with ranking), STC (with ranking), NSCR, and the number of publications cited more than 100 and 50 times. Developed countries, including six European countries, two North American countries, and one Oceania country, along with a developing country (China) occupied leading positions in MPs research. England led the NSC index and was second for STC, which indicated that it produced high quality MPs research. The USA also performed well in the areas of research depth and influence, as it ranked first for STC and had 62 publications that were cited more than 100 times, and 107 publications that were cited more than 50 times. China had the most publications (459), but ranked seventh for NSC. Canada, the other five Europe countries, and Australia also performed well. The 10 countries listed in Table 1 contributed 92.8% of all publications, and the high NSCR values (89.38% on average) indicated that these countries had great external influence not only in numbers of studies but also in the quality of MPs-related publications.

#### 3.1.3. Author Contribution Analysis

According to the statistics, 8191 authors (without debugging repetitions of authors’ names) have contributed to the increasing scientific knowledge about MPs. Table 2 lists the top 10 influential authors, ranked by the criteria of NSC on MPs issues, as well as their institution (the latest one), country, TNP, STC, and the number of ESI highly cited papers (NEHC). These data were manually debugged to improve the quality of analysis, as a single author may have different forms of abbreviations but with separately counted articles. Half of these influential authors are from European countries, which is in agreement with the known active participation of European countries in MPs-related research. Thompson, R.C. from England was the most productive and influential author, as his 48 publications were cited 11,617 times, and half of his publications were listed as ESI highly cited papers. Galloway, T.S. and Cole, M., from the University of Exeter, England, ranked second and fourth, respectively, with the ratio NEHC/TNP > 55%. Shi, H.H., who ranked ninth, was the most active MPs researcher in China, and 50% of his publications were included as ESI highly cited papers. Galgani, F.; Koelmans, A.A.; Thiel, M.; Rochman, C.M.; Shim, W.J. and Costa, M.F. also performed well with their MPs-related research, and contributed information about the MPs distribution in their regional marine environments, MPs analytical methods, hazardous chemical sorption of MPs, and release of MPs. These scholars were all listed as ESI highly cited researchers in the Ecology/Environment field, which indicated that their articles had significant influence on subsequent research.

#### 3.1.4. Journal Analysis

The 2637 publications were retrieved from 399 journals. Among them, most journals (384, 96.2%) published fewer than 20 articles about MPs. Table 3 shows the top 15 most productive journals in which more than 65% of publications related to MPs were published during the period 2004-2019. These journals were classified in six categories, and all placed in higher quartiles in category (Q1/Q2) according the 2018 JCR report. Ten of the journals were grouped in the *Environmental Sciences* category and three were grouped in the *Marine and Freshwater Biology* category, which indicated that MPs in the aquatic environment was the research hotspot. *Mar Pollut Bull* published the most articles (536) and had the highest STC and NSC, but its NSCR was lower than that of the other 14 journals. *Environ Pollut*, with 327 articles, ranked second. *Water Res* ranked eighth for TNP, but had the highest IF (7.913) among the ten *Environmental Sciences* journals. *Sci Rep-UK* and *Plos One*, were grouped in the *Multidisciplinary Sciences* category, and *Trac-Trend Anal Chem* and *Analmethods-UK* were grouped in the *Chemistry, Analytical* category. Articles in *Environ Toxicol Chem* and *Ecotox Environ Safe*, which were classified in the *Toxicology* category, focused on the ecotoxicological effects of MPs.

### 3.2. Network Analysis

#### 3.2.1. Co-Authorship Network Analysis of Countries

**Figure 2:**
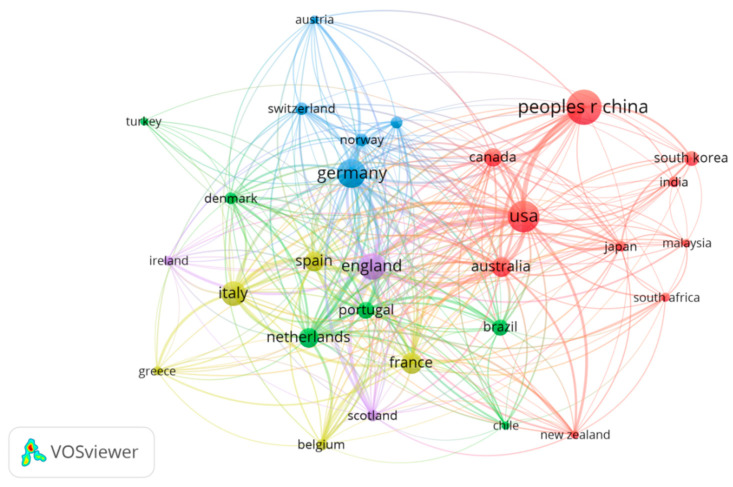
Co-authorship network diagram showing cooperation between countries (with a threshold of 30).

Figure 2 illustrates the collaboration network of countries conducting MPs research from 2004–2019. The number of publications threshold was set at 30, and of the 104 countries considered, 29 met this threshold. The whole network consisted of 29 nodes (referred to as countries) and 306 links (total link strength = 1815). England and the USA were the most affiliated countries; their close international cooperation was indicated by 28 links and a total link strength of 353 and 369, respectively. They were followed by Germany (links = 27, total link strength = 274), France (links = 26, total link strength = 218), and the Netherlands (links = 25, total link strength = 239). Academic collaboration between China and the USA was far more frequent than that of any other two countries (link strength = 52), which may be attributed to the high number of Chinese postgraduates/visiting scholars studying or working on MPs research in the USA. Other countries had fewer academic exchanges, such as Turkey (links = 9, total link strength = 12), possibly due to the consequence of language and finance barriers.

#### 3.2.2. Co-CitationNetwork Analysis of Cited References

**Figure 3:**
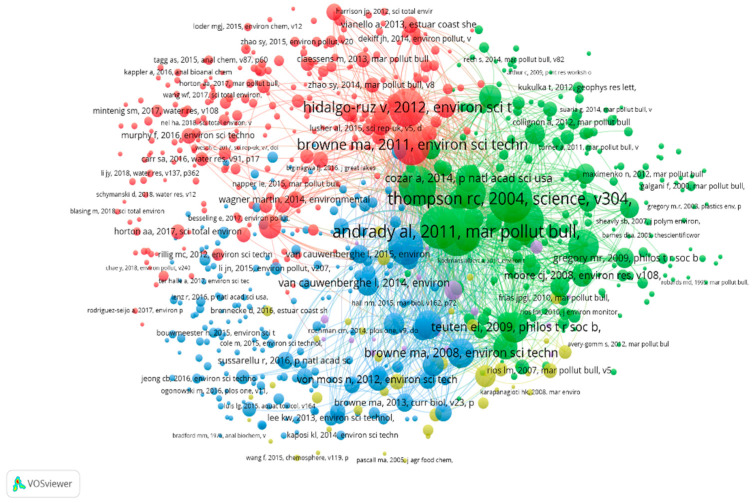
Co-citation network diagram of cited references from MPs articles cited a minimum of 30 times.

Of the 57,834 cited references from MPs articles published between 2004 and 2019, 713 references that were cited at least 30 times were used to create the co-citation network diagram (five clusters with different colors, Figure 3). Each cluster contained some core literatures with high citation rates and academic relationships, which revealed a knowledge base in the MPs research field.

In cluster red, the references with the largest nodes were the articles by Browne et al. [34] and Hidalgo-Ruz et al. [35], published in *Environ Sci Technol*, both with 712 co-citations and total link strengths of 25,800 and 24,418, respectively. Browne et al. [34] was the first study to explore the global distribution of MPs, which formed the knowledge base for MPs spatial distribution research. Hidalgo-Ruz et al. [35] reviewed 68 studies, to compare the methodologies used for MPs identification and quantification from seawater and sediment samples, and they called for standardized sampling programs to develop a more comprehensive understanding of MPs distribution. This study undoubtedly formed the knowledge base for MPs analytical methods. In cluster green, the documents with the largest nodes were authored by Andray [36] (published in *Mar. Pollut. Bull.*) and Thompson et al. [25] (published in *Science*). These articles were co-cited 712 times and had total link strengths of 29,817 and 26,743, respectively, indicating that they played a crucial role in the MPs co-citation network structure. Thompson et al. [25] clearly defined the term “MPs” and initiated global research on them. Andray [36] discussed the mechanism by which MPs are derived from marine debris, forming the knowledge base for MPs sources. In cluster blue, the document with the largest node (712 co-citations, 23,574 total link strength) was the article authored by Wright et al. [8] and published in *Environ Pollut.* Additionally, the laboratory experiments conducted by Setälä et al. [37] and Mattsson et al. [38] confirmed that MPs could transfer through food chains, and that lower trophic organisms could be the vector. In cluster yellow, the document with the largest node was written by Teuten et al. [39], who examined the uptake and subsequent release of hydrophobic organic contaminants present on plastic debris. This study formed a knowledge base about the interaction of MPs with contaminants. In cluster purple, Zettler et al. [40] first described a microbial community as a “plasticphere”, and called for research on the interaction between MPs and microorganisms.

#### 3.2.3. Co-Occurrence Network Analysis of Author Keywords

(1) In Publications Extracted from WoSCC

**Figure 4:**
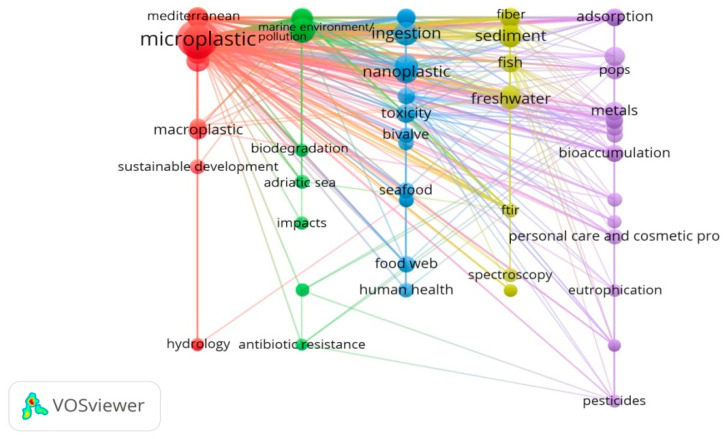
Co-occurrence network diagram of author keywords, appearing in a minimum of five publications between 2004 and 2019.

There were 4957 unique author keywords recorded in extracted publications from WoSCC. Among them, 3785 words (76.4%) were only used once, 566 (11.4%) were used twice, and 178 (3.6%) were used three times. These author keywords emphasized the breadth of MPs-related research, but also indicated a lack of continuity in research focuses. Some author keywords had different forms, but the same meaning (e.g., “FT-IR Spectroscopy” and “FT-IR” manually standardized as “FT-IR”), so we manually standardized 299 author keywords (with a minimum of 5 occurrences) to 230 keywords, and used them for co-occurrence network analysis (Figure 4). “Microplastic” (the biggest dot, Occurrence = 1146) was the most frequently used author keyword (and was used as our search term). The keywords “marine environment pollution” (occurrence = 366), “marine debris” (occurrence = 296), “ingestion” (occurrence = 105), “nanoplastic” (occurrence = 89), “sediments” (occurrence = 88), “polystyrene” (occurrence = 69), “FT-IR” (occurrence = 66), “freshwater” (occurrence = 60), and “polyethylene” (occurrence = 57) ranked second to tenth in the author keywords analysis, during the period from 2004 to 2019. These keywords were used in a large number of articles dealing with the distribution of MPs in different environments (e.g., marine environment, sediments, and freshwater), the ingestion of MPs by organisms, analytical techniques and quantification of these particles, and the biological effects of exposure to polystyrene or polyethylene nanoplastic. Figure 4 shows that the author keywords provided for MPs articles formed different clusters (by color), which represented global hotspots in MPs-related field (see Section 3.3).

(2) In ESI highly Cited Papers

**Figure 5:**
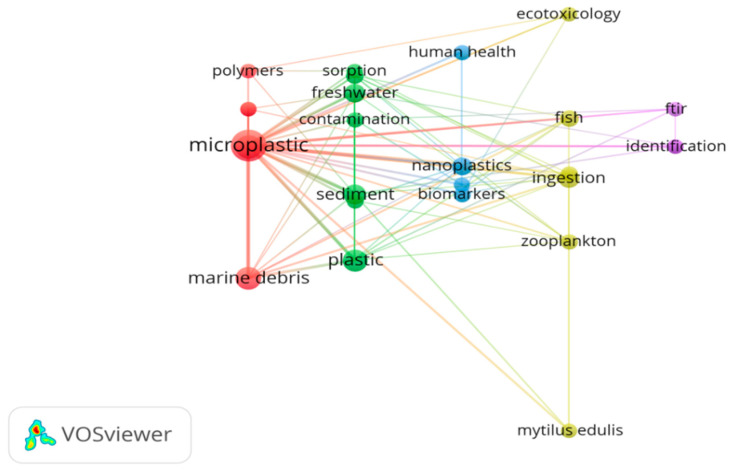
Co-occurrence network diagram of author keywords appearing in a minimum of five ESI highly cited papers, between 2010 and 2019.

There were 859 unique author keywords recorded in 395 ESI highly cited papers of MPs research, among which a total of 26 author keywords (manually standardized) appeared at least five times. The co-occurrence network analysis of these 26 author keywords were shown in Figure 5. “Microplastic” (the biggest dot, Occurrence = 198) was undoubtedly the most frequently used author keyword in ESI highly cited papers of MPs research, followed by “marine environment pollution” (occurrence = 72), “marine debris” (occurrence = 59), “sediments” (occurrence = 40), “ingestion” (occurrence = 28), “freshwater” (occurrence = 24), “nanoplastic” (occurrence = 23), “fish” (occurrence = 21), “accumulation” (occurrence = 13), “mussels” (occurrence = 12), ranked second to tenth. Most of these keywords were the same as those in publications extracted from WoSCC, and they also contributed to exploring the global hotspots of MPs-related research in the next section.

### 3.3. Hotspots of MPs Research

#### 3.3.1. MPs Sources and Spatial Distribution

Contributed keywords can be found in cluster red and green in Figure 4 and Figure 5, including marine plastic pollution, marine debris, beach, surface water, Mediterranean, accumulation, etc.

To address the key MPs problems, the international community has already developed policy responses, such as the European Marine Strategy Framework Directive, which is an ambitious program aimed at preventing MPs pollution. The scientific literature related to sources, pathways, and distribution of MPs is already substantial and constantly growing. MPs have been observed in almost every habitat of the aquatic environment, including ocean surface water [41], the water column [42], beaches [43], subtidal and deep-sea sediments [44,45], and freshwater lakes [46]. However, how to integrate MPs monitoring into existing environmental monitoring programs with reliable quantification routines requires further work.

#### 3.3.2. MPs Analytical Methods

Contributed keywords can be found in cluster yellow in Figure 4 and cluster purple in Figure 5, including identification, density separation, elutriation, sediment, spectroscopy, FT-IR, Raman, etc.

MPs analytical methods were developed to meet different purposes in MPs surveys, and can be divided into three steps: (1) sampling; (2) pretreatment for MPs extraction; and (3) qualitative and quantitative analysis. For sediment samples, high density solutions were commonly used to extract MPs, based on density separation [47]. In addition, elutriation columns proved to be useful tools with high extraction efficiency [48]. For seawater or water samples, selective sampling [49,50] and bulk sampling [51] methods were used in different studies, as were different pretreatment methods, such as enzyme digestion with subsequent filtration [49], and the sieve method [50]. For biota samples, the sampling methods always depended on the organisms being studied. For example, plankton trawls and nets were used to study the accumulation of MPs in plankton [52], whereas the dissection of different organs [53] was used for other target species. Digestion pretreatment methods for MPs extraction from biota samples have received a lot of attention, and many studies compared different digestion pretreatment methods, in pursuit of higher extraction efficiency [54]. For qualitative and quantitative analysis, MPs were usually measured and classified by shape, size, color, and chemical components. Most commonly, the extracted MPs were visually sorted under a microscope, and in some cases, the chemical components of MPs were determined by Raman spectroscopy [49], FT-IR micro-spectroscopy [51], and FT-IR [53]. It is notable that MPs analytical methods are now still debatable and have yet to be standardized.

#### 3.3.3. The Interaction of MPs with Contaminants

Contributed keywords can be found in cluster purple in Figure 4 and cluster green in Figure 5, including sorption, heavy metals, polychlorinated biphenyls (PCBs), polycyclic aromatic hydrocarbons (PAHs), phthalates, persistent organic pollutants (POPs), etc.

Partitioning of contaminations to MPs has also been well documented. It has been evidenced that POPs [55], such as PCBs and PAHs, as well as heavy metals, can be adsorbed onto the surface of MP particles [56], and the sorption capacity is not only influenced by external factors, such as salinity, environment temperature, and weathering, but also by polymer type [57]. Additionally, contaminants adsorbed to MPs as well as MPs additives (e.g., phthalates, alkylphenols) can be desorbed from the surface of MPs during their transit through the digestive tract in organisms [58], which may have negative impacts on them [59]. The challenges of understanding MPs sorption/desorption behavior in different environments and their combined toxic effects on organisms require further research.

#### 3.3.4. MPs Accumulation in Organisms and Their Potential Impact

Contributed keywords can be found in cluster blue in Figure 4 and cluster blue and yellow in Figure 5, including ingestion, biomarkers, mussel, fish, seafood, trophic transfer, food web, toxicity, human health, etc.

MPs have been detected in many wild aquatic organisms, such as beaked whale [53], lobster [60], crab [61], fish [62,63], bivalves [64,65,66], and zooplankton [52,67,68]. Laboratory experiments have shown that MPs can cause some physical harm to a diverse array of organisms upon ingestion [8]. Additional threats of MPs are their capacity to leach toxic additives such as monomers and plasticizers, and to be potential vectors for hydrophobic POPs, which may cause further health problems, such as endocrine disruption and even carcinogenesis to organisms upon ingestion [69]. To quantify the exposure risk from ingested MPs and to evaluate theirpotential eco-toxicological risk, scientists have studied the survival rate [70,71], growth [72], reproductive status [73,74] and gene expression [75] of target species. As biomarkers are useful indicators of exposure, they should be used to identify ecologically significant effects of MPs on sentinel species.

In recent years, the global presence of MPs and nanoplastics in foodstuff, drinking water, and air samples has been well documented. Consequently, human exposure to MPs via ingestion and inhalationis inevitable. Initial concern about human exposure to MPs focused on ingestion of MPs contaminated aquatic organisms. Evidence has shown that the consumption of entirely consumed organisms such as oysters and mussels may pose a higher risk of MPs exposure than consumption of eviscerated ones [76]. Other sources of human exposure to MPs include commercial salt and bottled drinks, as well as airborne MPs that can be inhaled [77]. Although MPs have been detected in human stool samples [78], nanoplastics reportedly might decrease the viability of human Caco-2 cells [79], and they can induce pro-inflammatory responses [80]—adverse effects of MPs on human health have not been reported to date. Thus, studies of the effects of MPs on human health are still urgently needed.

## 4. Conclusions

MPs, as an environmental pollutant, have become a global problem and they may pose a risk to human health. To summarize research progress and identify future research topics based on current hotspots, we conducted a bibliometric profile of MPs relevant research, using data from the WoSCC database for the period 2004–2019. We found that the scientific output of MPs-related research experienced rapid growth during the past 16 years, and that this booming research area has expanded into many related fields. Developed countries were important contributors to MPs research, as England, the USA, and Germany occupied the top three positions, based on the criterion of NSC. China was the only developing country in the top 10 national contributors. These influential countries foster close academic collaborations, as shown by the co-authorship network analysis of countries. However, more exchanges and cooperation between these countries and others are needed. We also found that Thompson RC, who defined the term “MPs”, was the most productive author, as well as the most influential one. All of the top 10 authors identified based on the criterion of NSC were ESI highly cited researchers in the *Ecology/Environment* field, which highlights their significant influence on MPs-related research. Our results show that the issue of MPs is a multidisciplinary research field, because journals classified in six different categories contained MPs-related articles. The results of co-citation network analysis of cited references indicated that in-depth research laid a solid foundation for the MPs scientific field. The internal composition relationships of MPs studies were visualized by co-occurrence networks of author keywords, both in extracted publications and ESI highly cited papers, which identified the following research hotspots: potential sources and spatial distributions of MPs, analytical methods, the interaction of MPs with contaminants, and the impacts of MPs on organisms as well as human beings. Future MPs studies should focus on the following five aspects: (1) integration of MPs monitoring into existing environmental monitoring programs; (2) unified technical standards and reliable quantification routines; (3) sorption/desorption behavior of contaminants on MPs in different environments; (4) biological effects on sentinel species and molecular toxicology mechanisms; and (5) the effects of MPs exposure on human health.

## Figures and Tables

**Table 1:** The top 10 countries for MPs research ranked by NSC; values for other criteria are given as well.

Rank	Country	NSC ^1^	TNP (R) ^2^	STC (R) ^3^	NSCR ^4^	≥100 ^5^	≥50 ^6^
1	England	23,182	282 (4)	25,376 (2)	91.3%	60	101
2	USA	23,166	381 (2)	25,472 (1)	90.9%	62	107
3	Germany	11,187	317 (3)	127,28 (3)	87.9%	37	76
4	France	10,015	179 (6)	107,83 (4)	92.9%	29	51
5	Netherlands	8369	157 (8)	9096 (6)	92.0%	30	52
6	Australia	8001	155 (9)	8496 (7)	94.2%	21	36
7	China	7491	459 (1)	10,315 (5)	72.6%	19	54
8	Canada	6050	129 (10)	6393 (8)	94.6%	17	28
9	Spain	5084	159 (7)	5504 (9)	92.4%	13	27
10	Italy	4703	230 (5)	5530 (10)	85.0%	15	28

^1^ NSC: Non-self-citation; ^2^ TNP (R): Total number of publications (ranking); ^3^ STC(R): Sum of times cited (ranking).^4^ NSCR: Non-self-citation ratio; ^5^ ≥100: the number of publications cited more than 100 times; ^6^ ≥50: the number of publications cited more than 50 times.

**Table 2:** The top 10 authors for MPs research ranked by NSC; values for other criteria are given as well.

Rank	Author	Organization	Country	NSC	TNP	STC	NEHC ^1^
1	Thompson, R.C.	University of Plymouth	England	11,371	48	11,617	24
2	Galloway, T.S.	University of Exeter	England	6616	31	6739	18
3	Galgani, F.	Ifremer ^2^	France	4038	28	4110	10
4	Cole, M.	University of Exeter	England	3100	20	3178	11
5	Koelmans, A.A.	Wageningen University	Netherlands	2988	39	3173	12
6	Thiel, M.	Universidad Catolica del Norte	Chile	2570	18	2612	5
7	Rochman, C.M.	University of Toronto	Canada	1654	24	1696	5
8	Shim, W.J.	KIOST ^3^	SouthKorea	1629	33	1767	5
9	Shi, H.H.	East China Normal University	China	1615	36	1761	18
10	Costa, M.F.	Federal University of Pernambuco	Brazil	1277	20	1347	1

^1^ NEHC: Number of ESI highly cited papers; ^2^ Ifremer: Institut Français de Recherche pour I’Exploitation de la Mer; ^3^ KIOST: Korea Institute of Ocean Science Technology.

**Table 3:** The top 15 productive journals that published articles about the MPs issue.

Rank	Journal	TNP	STC(R)	NSC(R)	NSCR	IF	Categories (Quartile)
1	Mar Pollut Bull	536	22,572(1)	18,544(1)	82.2%	3.782	Environmental Sciences (Q2); Marine and Freshwater Biology (Q1)
2	Environ Pollut	327	14,456(3)	12,711(3)	87.9%	5.714	Environmental Sciences (Q1)
3	Sci Total Environ	206	6082(4)	5534(4)	91.0%	5.589	Environmental Sciences (Q1)
4	Environ Sci Technol	172	14,466(2)	13,580(2)	93.9%	7.149	Environmental Sciences (Q1)
5	Chemosphere	90	2139(9)	2036(9)	95.2%	5.108	Environmental Sciences (Q1)
6	Environ Sci Pollut R	63	867(11)	835(11)	96.3%	2.914	Environmental Sciences (Q2)
7	Sci Rep-UK	63	3134(5)	3071(5)	98.0%	4.011	Multidisciplinary Sciences (Q1)
8	Water Res	49	2583(6)	2424(7)	93.8%	7.913	Environmental Sciences (Q1); Water resources (Q1)
9	Front Mar Sci	31	435(14)	425(14)	97.7%	3.086	Marine and Freshwater Biology (Q1)
10	Mar Environ Res	29	2467(7)	2431(6)	98.5%	3.445	Environmental Sciences (Q2); Marine and Freshwater Biology (Q1)
11	Trac-Trend Anal Chem	29	563(13)	551(13)	97.9%	8.428	Chemistry, Analytical (Q1)
12	Analmethods-UK	28	749(12)	739(12)	98.7%	2.378	Chemistry, Analytical (Q2)
13	Environ Toxicol Chem	28	1127(10)	1094(10)	97.0%	3.421	Environmental Sciences (Q2); Toxicology(Q2)
14	PlosOne	25	2430(8)	2399(8)	98.7%	2.776	Multidisciplinary Science(Q2)
15	Ecotox Environ Safe	21	304(15)	298(15)	98.0%	4.527	Environmental Sciences (Q1); Toxicology(Q1)
